# Changes in the Activities of Daily Living and Associated Factors of Older Adults After Home‐Based Rehabilitation: A Multicenter Prospective 6‐Month Follow‐Up Study

**DOI:** 10.1002/jgf2.70124

**Published:** 2026-05-15

**Authors:** Shogo Kawada, Takami Maeno, Shoji Yokoya, Tetsuhiro Maeno

**Affiliations:** ^1^ Woonzorgcentrum Nijenstede Beweging 3.0 Amersfoort the Netherlands; ^2^ Research and Development Center for Lifestyle Innovation University of Tsukuba Tsukuba Ibaraki Japan; ^3^ Center for Medical Education The Jikei University School of Medicine Tokyo Japan; ^4^ Department of Family Medicine, General Practice and Community Health, Institute of Medicine University of Tsukuba Tsukuba Ibaraki Japan; ^5^ Kitaibaraki Center for Family Medicine Kitaibaraki Ibaraki Japan

**Keywords:** changes in activities of daily living, cognitive function, follow‐up after home‐based rehabilitation

## Abstract

**Background:**

Older adults may experience changes in activities of daily living (ADL) after completing home‐based rehabilitation; however, no evidence exists regarding post‐rehabilitation outcomes and the factors influencing long‐term changes in ADL. This study aimed to investigate changes in ADL in older adults following home‐based rehabilitation and identify factors associated with these changes.

**Methods:**

This multicenter prospective cohort study included older adults aged ≥ 65 years who had completed home‐based rehabilitation. We assessed ADL at the completion of rehabilitation and 6 months later. Subsequently, we used multiple regression analysis to examine factors associated with changes in ADL.

**Results:**

In total, 49 participants were included in the analysis. Six months after home‐based rehabilitation, ADL improved or was maintained in 38 participants (77.6%), and declined in only 11 participants (22.4%) compared with those immediately after home‐based rehabilitation. Multiple regression analysis showed that lower cognitive function was significantly associated with a decline in ADL at 6 months after home‐based rehabilitation (*β* = −0.387, *p* = 0.005).

**Conclusions:**

Continued monitoring and support for older adults with lower cognitive function after completing home‐based rehabilitation may be necessary to prevent declines in ADL.

## 1 Introduction

In Japan, the increasing number of care‐dependent older adults has caused serious social issues, such as increasing pressure on social security expenditures and a shortage of rehabilitation personnel [1]. Supporting the health and independence of this population is essential, but human and financial resources are limited. Recently, home‐based rehabilitation has increasingly gained attention as it allows time‐limited goals, such as improvements in activities of daily living (ADL) and instrumental activities of daily living (IADL), as well as the achievement of social participation, to be set and can be concluded once these goals are achieved [2, 3], allowing resources to be reallocated to address emerging demands, such as other older adults newly requiring rehabilitation services.

Despite achieving their goals, many older adults receiving home‐based rehabilitation have permanent disabilities or limitations in ADL, requiring continued care. The ADLs of those discharged from rehabilitation may subsequently decline [4, 5]. Although being discharged is a key objective, preventing functional deterioration thereafter is equally important. Thus, appropriate prognostic evaluation and adequate support are essential when planning to complete home‐based rehabilitation.

Several studies have examined longitudinal changes in ADL among older adults who need long‐term care [6, 7, 8]. Approximately 31% of care‐dependent older adults in nursing homes [6] and 30%–40% of those living at home [7, 8] experience declines in ADL within 6 months, even without acute events such as hospitalization or the occurrence of new diseases. In addition, cognitive decline is a known predictor of ADL decline in older adults, including those who are care‐dependent. Poorer ADL recovery has been observed in older adults with dementia in post‐acute rehabilitation wards [9], and similarly, a greater ADL decline over 6 months has been reported in care‐dependent nursing home residents with moderate to severe cognitive impairment [6]. In addition, ADL decline has been associated with poorer physical function [10], poorer mental health [11, 12], and increased social isolation [13]. However, no studies have examined changes in ADL and their associated factors after the completion of home‐based rehabilitation.

Clarifying such changes in ADL and related factors may aid appropriate decision‐making regarding rehabilitation termination and inform targeted interventions to prevent ADL decline in high‐risk older adults, thereby promoting independence.

This study aimed to examine changes in ADL after the completion of home‐based rehabilitation and identify background factors associated with those changes.

## 2 Methods

### 2.1 Setting and Participants

We conducted a multicenter prospective cohort study at the home‐based rehabilitation departments of four general hospitals located in Aichi, Gifu, Nara, and Hiroshima prefectures, Japan. In Japan, home‐based rehabilitation requires a physician's prescription. This service is mainly funded by the government's Long‐Term Care Insurance system. The service volume is determined by the user's care‐need level, though it is typically provided once or twice a week for 40 to 60 min per session. It primarily focuses on practical, ADL‐oriented exercises, environmental modifications, family education, and self‐training guidance. At each institution, home‐based rehabilitation was designed to conclude upon achieving intervention goals whenever possible. At the start of care, individualized goals and intervention duration were set, and service users were informed that rehabilitation could be concluded upon goal attainment. They also received the *Ikō Shien Kasan* (Transition Support Incentive in Home‐Based Rehabilitation), a Japanese incentive system that evaluates and rewards home‐based rehabilitation services when improvements in ADL or IADL enable clients to transition to other care services.

This study enrolled older adults aged ≥ 65 who had completed home‐based rehabilitation at these institutions between June 1, 2020, and October 31, 2021. The exclusion criteria included progressive diseases (such as neurodegenerative disorders and cancer), termination of rehabilitation due to death, hospitalization, institutionalization, medical reasons, lack of consent, or inability to understand or respond to the questionnaire.

### 2.2 Data Collection

We collected data at the completion of home‐based rehabilitation and 6 months thereafter. Assessments were conducted by the physical or occupational therapists who provided rehabilitation at each institution. Before the study, the first author provided verbal and written instructions to all assessors regarding the study purpose, outcome measures, and assessment methods.

At the completion of home‐based rehabilitation, we investigated the following: participant characteristics (age, sex, primary diagnosis, comorbidities, presence of a spouse, number of cohabitants, educational background, and care‐need level); details of home‐based rehabilitation services (duration and frequency of rehabilitation, time per session, and achievement of social participation goals); health and function (ADL, self‐rated health [14], cognitive function, mental health, and social support/network). We assessed comorbidities, ADL, cognitive function, mental health, and social support/networks using the Charlson Comorbidity Index (CCI) [15], Barthel Index (BI) [16], Mini‐Cog [17], Patient Health Questionnaire‐2 (PHQ‐2) [18], and Japanese version of the Lubben Social Network Scale, short form (LSNS‐6), respectively [19]. Briefly, the CCI measures the burden of comorbid conditions, with higher scores indicating greater severity. The BI measures the older adult's ability to perform ADL such as toileting and showering, with a score ranging from 0 to 100, where 100 indicates a high level of independence. The Mini‐Cog screens for cognitive impairment (score 0–5; higher scores indicate better cognitive function). The PHQ‐2 screens for depressive symptoms (score 0–6; higher scores indicate higher risk). The LSNS‐6 measures social network strength (score 0–30; higher scores represent stronger networks and lower isolation risk). Six months after the completion of home‐based rehabilitation, we investigated the occurrence or causes of hospitalization, institutionalization, or death and reassessed ADL. The same evaluator conducted a semi‐structured telephone interview with the participant or primary caregiver.

### 2.3 Data Analysis

#### 2.3.1 Participants Included in the Analysis

For the analysis, we only included participants who completed ADL assessment at home 6 months after the completion of home‐based rehabilitation and excluded those who were hospitalized or received reintervention during the follow‐up period, as missing 6‐month data precludes the calculation of ADL changes and hospitalization or reintervention could substantially affect ADL, obscuring its natural course.

#### 2.3.2 Changes in ADL 6 Months After the Completion of Home‐Based Rehabilitation

We assessed the normality of continuous variables using the Shapiro–Wilk test and applied non‐parametric tests for non‐normally distributed variables. We used a Wilcoxon signed‐rank test to analyze changes in ADL scores between those at the completion of home‐based rehabilitation and those at 6 months after. The distribution and breakdown of ADL scores were described using descriptive statistics. ADL changes were classified as “improved,” “maintained,” or “declined.” We presented the distribution as absolute and relative changes; the latter were calculated by dividing the absolute change by the baseline ADL at the completion of home‐based rehabilitation [20].

#### 2.3.3 Factors Associated With Changes in ADL at 6 Months After the Completion of Home‐Based Rehabilitation

The relative change in ADL at 6 months after the completion of home‐based rehabilitation was used as the outcome, which was calculated by dividing the absolute change by the baseline ADL score to adjust for variability in baseline ADL among participants [20].

Age, CCI, number of cohabitants, duration and frequency of rehabilitation, time per session, BI, and LSNS‐6 were treated as continuous variables; all others were treated as categorical. Educational background was divided into four categories: “junior high school,” “high school,” “vocational school/junior college,” and “university and above.” These were subsequently dichotomized into two groups: “junior high school” and “high school or above.” We classified care‐need levels into three groups: “support levels 1–2,” requiring assistance with IADL such as cleaning or cooking despite stable mobility; “care‐need levels 1–3,” requiring assistance with basic mobility such as walking and rising; and “care‐need levels 4–5,” requiring extensive assistance with overall ADL. In addition, we dichotomized self‐rated health into “not healthy” (“not healthy” and “somewhat not healthy”) and “healthy” (“somewhat healthy” and “healthy”). Mini‐Cog scores ≤ 2 were defined as “dementia present” and ≥ 3 as “dementia absent.” PHQ‐2 scores ≥ 3 were categorized as “depression present” and ≤ 2 as “depression absent.”

Initially, we conducted univariate analyses to examine associations between each variable and the relative change in ADL at 6 months after the completion of home‐based rehabilitation. For these analyses, we used Mann–Whitney *U* tests for two‐group categorical variables, Kruskal–Wallis tests for those with ≥ three groups, and Spearman's rank correlation for continuous variables. Subsequently, we conducted multivariate analysis. Given the exploratory nature of this study, we selected variables with a *p*‐value < 0.1 or a correlation coefficient ≥ 0.3 [21] in the univariate analyses. These variables, along with age and sex, were entered as independent variables in a forced‐entry multiple linear regression, with the relative change in ADL as the dependent variable. To address multicollinearity, we included only one of the strongly correlated variables (*p* < 0.1 or *ρ* ≥ 0.3) identified in univariate analyses. Strong associations were defined as correlation coefficients ≥ 0.7 for continuous variables. For categorical variables, we used chi‐square or Mann–Whitney *U* tests, with *p* < 0.05 considered statistically significant.

We calculated the sample size based on the planned multiple regression analysis. Assuming we would include five explanatory variables in the model and applying the rule of thumb of 10 participants per variable, we estimated the required sample size for the analysis to be 50.

All analyses were performed using SPSS version 25.0 for Mac, with a significance level of 5%.

### 2.4 Ethical Considerations

This study was approved by the Medical Ethics Committee of the University of Tsukuba (No. 2820) and the ethics committees of all participating institutions. Written informed consent was obtained from all participants after providing verbal and written explanations.

## 3 Results

### 3.1 Participant Characteristics at the Completion of Home‐Based Rehabilitation

Approximately 64 individuals completed home‐based rehabilitation during the study period; 15 were excluded owing to follow‐up events, leaving 49 for analysis. Among those excluded from the analysis, 14 were due to attrition (three died and 11 were hospitalized) and one was due to loss to follow‐up (could not be contacted). Table 1 summarizes the characteristics of the participants included in the analysis. Their mean age was 80.1 ± 7.1 years (range: 67–91), and 37 (75.5%) were female. Musculoskeletal disorders were the most common cause of home‐based rehabilitation (36 participants, 73.5%), followed by cerebrovascular disorders (nine participants, 18.4%).

**TABLE 1:** Participant characteristics (*n* = 49).

Variable	
Age, mean ± SD (years)	80.1 ± 7.1
Sex, Female, *n* (%)	37 (75.5)
Diagnosis category, *n* (%)	
Cerebrovascular	9 (18.4)
Musculoskeletal	36 (73.5)
Cardiovascular	1 (2.0)
Respiratory	0
Other	3 (6.1)
BI (points), median [IQR]	95.0 [85.0, 100.0]
CCI (points), median [IQR]	0.0 [0.0, 2.0]
Spouse present, *n* (%)	38 (77.6)
Educational background, *n* (%)	
Junior high school	15 (32.6)
High school	18 (39.1)
Vocational school/Junior college	10 (21.7)
University and above	3 (6.5)
Care‐need level, *n* (%)	
Support level 1	4 (8.2)
Support level 2	16 (32.7)
Care‐need level 1	5 (10.2)
Care‐need level 2	11 (22.4)
Care‐need level 3	6 (12.2)
Care‐need level 4	7 (14.3)
Care‐need level 5	0
Duration of home‐based rehabilitation (days), median [IQR]	80.0 [56.0, 98.0]
Distribution of duration, *n* (%)	
< 1 month	4 (8.2)
< 3 months	32 (65.3)
< 6 months	9 (18.4)
< 12 months	4 (8.2)
Frequency of home‐based rehabilitation (times/week), median [IQR]	1.0 [1.0, 2.0]
Distribution of frequency, *n* (%)	
Once every 2 weeks	1 (2.0)
Once a week	30 (61.2)
Twice a week	16 (32.7)
Three times a week	1 (2.0)
Other	1 (2.0)
Time per session (min/session), median [IQR]	60.0 [40.0, 60.0]
Distribution of time per session, *n* (%)	
40 min	21 (42.9)
60 min	28 (57.1)
Achievement of goals (multiple responses), *n* (%)	
Body function level	25 (51.0)
Activity level	45 (91.8)
Social participation level	13 (26.5)

*Note:* Continuous variables are expressed as mean ± SD for normally distributed data, and as median [IQR] for non‐normally distributed data.

Abbreviations: BI, Barthel index; CCI, Charlson comorbidity index; IQR, interquartile range; SD, standard deviation.

### 3.2 Changes in ADL at 6 Months After the Completion of Home‐Based Rehabilitation

**FIGURE 1:**
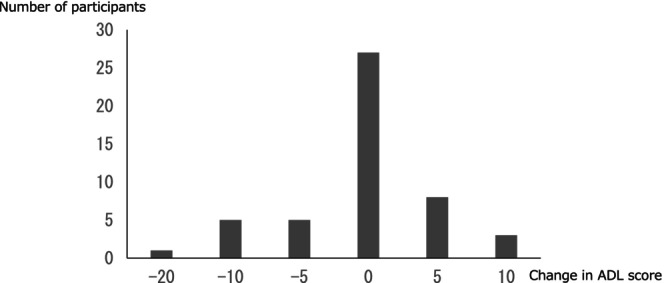
Distribution of absolute changes in ADL scores at 6‐month follow‐up. The bar graph illustrates the distribution of absolute changes in ADL scores at the completion of home‐based rehabilitation and at 6 months later: +10 points in three cases (6.1%), +5 points in eight cases (16.3%), 0 points in 27 cases (55.1%), −5 points in five cases (10.2%), −10 points in five cases (10.2%), and −20 points in one case (2.0%). ADL, activities of daily living.

The BI score was 95.0 [85.0, 100.0] at the completion of home‐based rehabilitation and 95.0 [80.0, 100.0] at 6 months, with no significant change. Compared with that at the completion of rehabilitation, ADL improved or was maintained in 38 participants (77.6%), and declined in 11 participants (22.4%) at 6 months. Figure 1 illustrates the detailed distribution of absolute changes in ADL scores at the completion of home‐based rehabilitation and at 6 months later. Similarly, the distribution of relative ADL changes was > 0.1 to 0.2 points in three cases (6.1%), > 0 to 0.1 points in eight cases (16.3%), 0 points in 27 cases (55.1%), < 0 to −0.1 points in five cases (10.2%), < −0.1 to −0.2 points in five cases (10.2%), and < −0.2 to −0.3 points in one case (2.0%).

### 3.3 Factors Associated With Changes in ADL After the Completion of Home‐Based Rehabilitation

Tables 2 and 3 present the results of univariate analyses of factors associated with relative ADL changes at 6 months after the completion of home‐based rehabilitation. Mann–Whitney *U* tests and Kruskal–Wallis tests revealed no significant differences based on sex, primary diagnosis, presence of a spouse, care‐need level, use of public home‐care services, achievement of social participation goals, PHQ‐2 scores, or self‐rated health. In contrast, a significant difference was observed in relative ADL changes between participants in the “junior high school” (0.000 [−0.081, 0.000]) and “high school or above” (0.000 [0.000, 0.026]; *p* = 0.038) groups, with ADL declining in the “junior high school” group. Similarly, a significant difference was observed in ADL changes between participants with and without cognitive impairment based on the Mini‐Cog (−0.026 [−0.091, 0.000] vs. 0.000 [0.000, 0.053], *p* = 0.015), indicating greater ADL decline in those with cognitive impairment. Correlation analyses revealed no significant associations between relative changes in ADL and age, CCI, number of cohabitants, duration and frequency of rehabilitation, LSNS‐6 scores, or ADL scores at the completion of home‐based rehabilitation. However, a significant negative correlation was observed between relative ADL changes and the time per session of home‐based rehabilitation (*ρ* = −0.318, 95% CI: −0.561 to −0.026, *p* = 0.026), indicating that a longer time per session was associated with less relative improvement in ADL.

**TABLE 2:** Factors associated with relative ADL change: Univariate analysis.

Variable	*n*	ADL at the completion of home‐based rehabilitation	ADL at 6 months after the completion of home‐based rehabilitation	Relative ADL change	*p* *
Sex					
Female	37	95.0 [90.0, 100.0]	95.0 [85.0, 100.0]	0.000 [0.000, 0.000]	0.340
Male	12	92.5 [77.5, 100.0]	97.5 [80.0, 100.0]	0.000 [0.000, 0.054]	
Primary diagnosis					
Cerebrovascular	9	95.0 [80.0, 100.0]	100.0 [85.0, 100.0]	0.000 [0.000, 0.053]	0.186
Musculoskeletal	36	95.0 [90.0, 100.0]	95.0 [90.0, 100.0]	0.000 [−0.050, 0.000]	
Cardiovascular	1	65.0	65.0	0.000	
Other	3	75.0 [72.5, 82.5]	75.0 [72.5, 87.5]	0.000 [0.000, 0.056]	
Spouse					
Present	38	95.0 [90.0, 100.0]	100.0 [90.0, 100.0]	0.000 [0.000, 0.053]	0.649
Not present	11	95.0 [77.5, 95.0]	95.0 [75.0, 97.5]	0.000 [0.000, 0.000]	
Educational Background					
Junior High School	15	95.0 [82.5, 100.0]	90.0 [75.0, 100.0]	0.000 [−0.081, 0.000]	0.038
High school or above	31	95.0 [90.0, 100.0]	100.0 [95.0, 100.0]	0.000 [0.000, 0.026]	
Care‐need levels					
Support levels 1–2	20	97.5 [95.0, 100.0]	100.0 [95.0, 100.0]	0.000 [0.000, 0.000]	0.936
Care‐need levels 1–3	22	92.5 [80.0, 100.0]	95.0 [75.0, 100.0]	0.000 [−0.050, 0.052]	
Care‐need levels 4–5	7	95.0 [77.5, 97.5]	100.0 [77.5, 100.0]	0.000 [−0.026, 0.026]	
Achievement of social participation goals					
Achieved	13	95.0 [90.0, 100.0]	100.0 [95.0, 100.0]	0.000 [0.000, 0.052]	0.414
Not achieved	36	95.0 [85.0, 100.0]	95.0 [80.0, 100.0]	0.000 [−0.025, 0.000]	
Mini‐Cog					
Dementia present	10	95.0 [75.0, 100.0]	82.5 [75.0, 95.0]	−0.026 [−0.091, 0.000]	0.015
Dementia absent	39	95.0 [90.0, 100.0]	100.0 [95.0, 100.0]	0.000 [0.000, 0.052]	
PHQ‐2					
Depression present	6	95.0 [90.0, 95.0]	95.0 [95.0, 95.0]	0.000 [0.000, 0.000]	0.459
Depression absent	43	95.0 [85.0, 100.0]	100.0 [80.0, 100.0]	0.000 [−0.025, 0.000]	
Self‐rated health					
Not healthy	17	95.0 [95.0, 100.0]	95.0 [95.0, 100.0]	0.000 [0.000, 0.053]	0.294
Healthy	32	95.0 [85.0, 100.0]	95.0 [75.0, 100.0]	0.000 [−0.051, 0.000]	

*Note:* Median [IQR].

Abbreviations: ADL, activities of daily living; PHQ‐2, patient health questionnaire‐2.

* Associations between relative ADL change and each variable were examined using Mann–Whitney *U* tests or Kruskal–Wallis tests.

**TABLE 3:** Factors associated with relative changes in ADL: Univariate analysis (correlation analysis).

Variable	Correlation coefficient	*p* *
Age (years)	−0.180	0.216
CCI (points)	−0.039	0.788
Number of cohabitants	−0.048	0.745
Duration of home‐based rehabilitation (days)	0.243	0.092
Frequency of home‐based rehabilitation (times/week)	0.132	0.365
Time per session (min)	−0.318	0.026
ADL at the completion of home‐based rehabilitation (points)	0.001	0.996
LSNS‐6 (points)	−0.106	0.469

Abbreviations: ADL, activities of daily living; CCI, Charlson comorbidity index; LSNS‐6, Lubben social network scale‐6.

* Associations between relative changes in ADL and each variable were examined using Spearman's rank correlation coefficient.

Table 4 presents the results of multivariate analysis of factors associated with relative ADL changes at 6 months after the completion of home‐based rehabilitation. In univariate analysis, educational background, Mini‐Cog scores, and time per session met the inclusion criteria (*p* < 0.1 or correlation ≥ 0.3). These three variables, along with age and sex, were examined. A chi‐squared test showed a significant association between educational background and Mini‐Cog scores (*p* = 0.001), with more cognitive impairment observed in the junior high school group—an association reported in previous studies [22]. Owing to this overlap, we excluded educational background and performed multiple regression with age, sex, Mini‐Cog scores, and time per session as the forced‐entry variables. We conducted ANOVA to test the regression coefficients and observed a significant result (*p* = 0.014), with a coefficient of determination (*R* ^2^) of 0.173. The Durbin–Watson statistic was 1.714, indicating random errors, and no outliers beyond ±3 standard deviations were detected. Cognitive function (*β* = −0.387, *p* = 0.005) was significantly associated with relative ADL changes at 6 months after the completion of home‐based rehabilitation, with lower cognitive function related to a decline in ADL at that time point. Additionally, time per session showed a marginally significant negative association with relative ADL changes (*β* = −0.258, *p* = 0.057).

**TABLE 4:** Factors associated with relative changes in ADL: Multivariate analysis.

Variable	Standardized coefficient (*β*)	*p* *
Age (years)	−0.047	0.733
Sex (male = 0, female = 1)	−0.137	0.320
Time per session (min)	−0.258	0.057
Mini‐Cog (dementia absent = 0, dementia present = 1)	−0.387	0.005

Abbreviation: ADL, activities of daily living.

* We examined the associations between relative changes in ADL and each variable using multiple regression analysis.

## 4 Discussion

Approximately 20% of participants who completed home‐based rehabilitation showed a decline in ADL 6 months later, whereas 80% of participants exhibited a maintained or improved ADL. Multivariate analysis revealed that lower cognitive function was associated with a higher likelihood of ADL decline at that time point.

No statistically significant change was observed in ADL 6 months after the completion of home‐based rehabilitation. The ADL scores of 78% of the participants improved or were maintained, whereas 22% declined. Previous studies across various care settings—including patients immediately post‐discharge [23], care‐dependent older adults living at home [7, 8, 24], and those in nursing homes [6]—have consistently reported that approximately 30%–40% of participants experience ADL deterioration over a 4‐ to 12‐month follow‐up period. Compared to these reports, our findings demonstrated a slightly lower proportion of participants with decreased ADL. We attribute this lower deterioration rate to two main reasons. First, the participants had a very high functional capacity at the completion of home‐based rehabilitation; their median BI score was 95, indicating a very high level of independence [16]. A previous national survey in Japan supports this, reporting that among participants who completed home‐based rehabilitation within 6 months, 79% already had a BI of ≥ 80 at the start of the service [25]. Second, the participants had successfully completed a targeted rehabilitation program within their actual living environments. Having already adapted to their homes and optimized their daily movements, they likely possessed sufficient physical and functional reserve to prevent functional decline after completing the service.

Multivariate analysis showed that lower cognitive function was significantly associated with decreased ADL at 6 months after the completion of home‐based rehabilitation. This finding aligns with those of previous studies reporting associations between cognitive function and ADL recovery or prognosis. Specifically, moderate to severe cognitive impairment or dementia limits ADL recovery following acute events as observed in stroke survivors and patients with proximal femoral fractures [9, 26, 27]. Furthermore, cognitive impairment serves as a significant risk factor for long‐term ADL deterioration among care‐dependent older adults in nursing homes [6]. Therefore, low cognitive function reduces the effectiveness of rehabilitation and contributes to long‐term declines in physical function and ADL. Cognitive function is an important factor to consider when setting rehabilitation goals, planning treatment, and predicting prognosis. Our results confirmed that this relationship also persists beyond the completion of home‐based rehabilitation. After home‐based rehabilitation, older adults should actively maintain physical function and ADL rather than depending on therapist interventions. However, those with low cognitive function may struggle with self‐management, which may lead to ADL deterioration. Therefore, older adults with low cognitive function need adequate support to regularly evaluate their ADL and physical activity after the completion of home‐based rehabilitation, with timely care adjustments and family guidance.

This study had some limitations. First, potential confounding factors may not have been fully controlled. Second, our sample size was relatively small compared to typical prognosis studies, which generally include over 100 participants. Because of this small sample size, the lack of significant associations between changes in ADL at 6 months after the completion of home‐based rehabilitation and known factors may reflect insufficient statistical power, requiring larger samples from more institutes. Third, since participants had relatively high ADL and independence at the completion of rehabilitation, the results may not be generalizable to less independent older adults. Further studies with larger, functionally stratified samples are needed. Despite these limitations, a major strength of this study is its prospective design, which allowed for the systematic collection of data from older adults and minimized the risk of missing data.

In conclusion, approximately 20% of participants who completed home‐based rehabilitation showed a decline in ADL at 6 months. Multivariate analysis indicated that lower cognitive function was associated with ADL decline. For these older adults, regular monitoring of ADL and physical activity after the completion of rehabilitation and provision of appropriate support to adjust care services and guide families are essential.

## Author Contributions

**Shogo Kawada:** conceptualization, methodology, software, data curation, investigation, validation, formal analysis, supervision, funding acquisition, visualization, project administration, resources, writing – original draft, writing – review and editing. **Shoji Yokoya:** conceptualization, investigation, writing – original draft, writing – review and editing, visualization, methodology, validation, software, formal analysis, resources, supervision, data curation. **Tetsuhiro Maeno:** conceptualization, investigation, writing – original draft, writing – review and editing, visualization, validation, methodology, software, formal analysis, data curation, supervision, resources. **Takami Maeno:** conceptualization, methodology, software, data curation, investigation, validation, formal analysis, supervision, visualization, resources, writing – original draft, writing – review and editing.

## Funding

The authors have nothing to report.

## Ethics Statement

We conducted the study in accordance with the Declaration of Helsinki and with the approval of the University of Tsukuba Medical Ethics Board (no. 2820).

## Consent

All participants were informed about the study in writing. They provided written informed consent prior to enrollment in the study.

## Conflicts of Interest

The authors declare no conflicts of interest.

## Data Availability

The datasets generated and analyses performed as part of the current study are not publicly available due to the consent requirements of the participants. However, data on participants' characteristics, including sex‐ and age‐stratified descriptive data, are available from the corresponding author upon reasonable request.
